# Rapid determination of leaf area and plant height by using light curtain arrays in four species with contrasting shoot architecture

**DOI:** 10.1186/1746-4811-10-9

**Published:** 2014-04-11

**Authors:** Dimitrios Fanourakis, Christoph Briese, Johannes FJ Max, Silke Kleinen, Alexander Putz, Fabio Fiorani, Andreas Ulbrich, Ulrich Schurr

**Affiliations:** 1 IBG-2: Plant Sciences, Institute for Bio- and Geosciences, Forschungszentrum Jülich, 52425 Jülich, Germany; 2 Present address: Center of Applied Biology, Department of Soil Science and Plant Nutrition, Hochschule Geisenheim University, 65366 Geisenheim, Germany; 3 University of Applied Sciences Osnabrück, 49076 Osnabrück, Germany

**Keywords:** Leaf area estimation, Maximum height estimation, Overlapping plants, Phenotyping, Sensor speed, Sensor-to-plant system, Shoot profile, Shoot silhouette

## Abstract

**Background:**

Light curtain arrays (LC), a recently introduced phenotyping method, yield a binary data matrix from which a shoot silhouette is reconstructed. We addressed the accuracy and applicability of LC in assessing leaf area and maximum height (base to the highest leaf tip) in a phenotyping platform. LC were integrated to an automated routine for positioning, allowing *in situ* measurements. Two dicotyledonous (rapeseed, tomato) and two monocotyledonous (maize, barley) species with contrasting shoot architecture were investigated. To evaluate if averaging multiple view angles helps in resolving self-overlaps, we acquired a data set by rotating plants every 10° for 170°. To test how rapid these measurements can be without loss of information, we evaluated nine scanning speeds. Leaf area of overlapping plants was also estimated to assess the possibility to scale this method for plant stands.

**Results:**

The relation between measured and calculated maximum height was linear and nearly the same for all species. Linear relations were also found between plant leaf area and calculated pixel area. However, the regression slope was different between monocotyledonous and dicotyledonous species. Increasing the scanning speed stepwise from 0.9 to 23.4 m s^−1^ did not affect the estimation of maximum height. Instead, the calculated pixel area was inversely proportional to scanning speed. The estimation of plant leaf area by means of calculated pixel area became more accurate by averaging consecutive silhouettes and/or increasing the angle between them. Simulations showed that decreasing plant distance gradually from 20 to 0 cm, led to underestimation of plant leaf area owing to overlaps. This underestimation was more important for large plants of dicotyledonous species and for small plants of monocotyledonous ones.

**Conclusions:**

LC offer an accurate estimation of plant leaf area and maximum height, while the number of consecutive silhouettes that needs to be averaged is species-dependent. A constant scanning speed is important for leaf area estimations by using LC. Simulations of the effect of varying plant spacing gave promising results for method application in sets of partly overlapping plants, which applies also to field conditions during and after canopy closure for crops sown in rows.

## Background

The capacity to quantitatively explore plant phenotypes (from single cells to the whole plant) and their dynamic responses to a changing environment is a necessary requirement for genetic and physiological research by crop breeders, agricultural industry, and academia. Although molecular profiling technologies now enable the generation of a large amount of data with decreasing costs, the understanding of the link between genotype and phenotype still remains fragmented [1]. Insufficient technical and conceptual capacity of the plant scientific community to probe existing genetic resources and unravel environmental effects limits faster progress in this field [2]. To address this challenge, several automated phenotyping platforms have been developed from academia or commercial sources during the past decade (reviewed by [3-5]). The development of phenotyping applications for non-invasive assessment of the dynamics of plant biomass development is a cornerstone for this effort.

**Figure 1:**
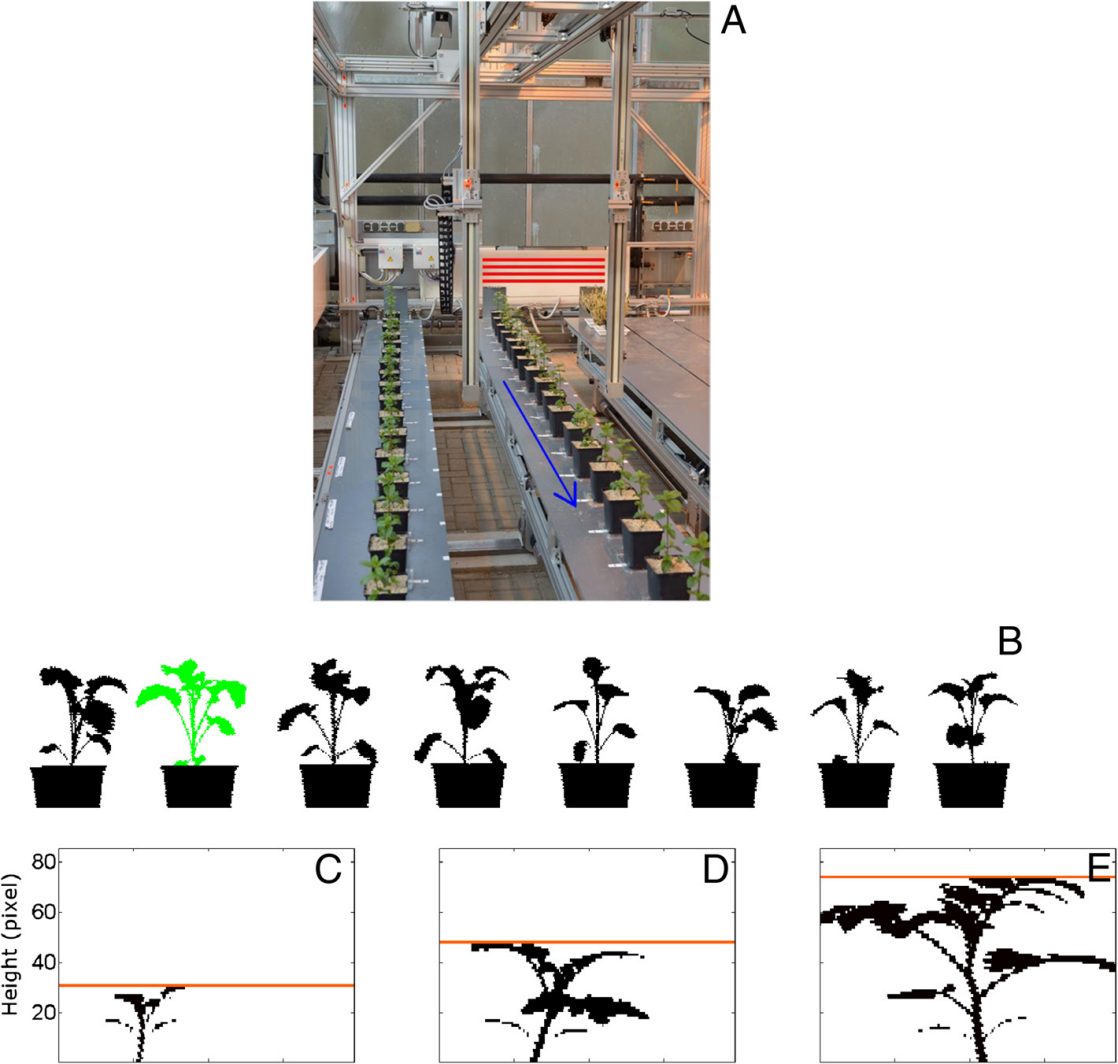
**Working principle of light curtain arrays (LC) and shoot silhouette extraction. (A)** LC are scanning a row of plants. The light barriers, arranged on the two vertical poles, emit and receive light beams (examples of which are shown by the red horizontal lines). The blue arrow depicts the movement of LC during the scan. Pot height is 13 cm. **(B)** Plant profile of a row of rapeseed plants. The green colour indicates the segmentation step during which the plant silhouette is separated from the pot silhouette. **(C–E)** Estimation of maximum height (base to the highest leaf tip; depicted by the vertical line) from the silhouette of tomato plants differing in size.

**Figure 2:**
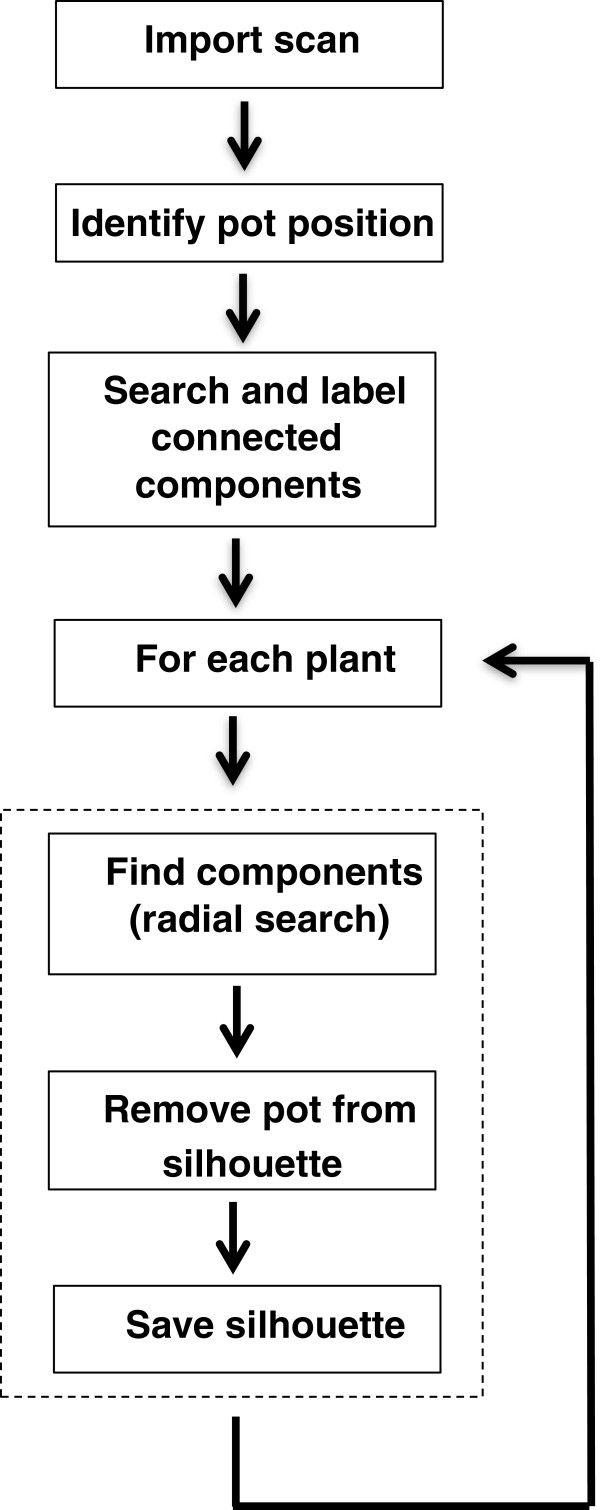
**Flow diagram of the data processing pipeline employed to extract a single shoot silhouette from a scan of several plants (see Figure **1**B).**

In phenotyping platforms the most commonly used method of assessing shoot biomass is by acquiring digital images of the plants, following their positioning at a specified orientation towards a camera under defined illumination conditions [1,6,7]. Following image acquisition, digital image processing enables the extraction of plant features from the image background based on colour and brightness analysis [8]. The main limitations of biomass assessment by using imaging methods such as colour imaging in 2D spatial dimensions are: a) overlapping leaves and stems lead to underestimation of shoot area and often restrict this application to a given plant size or developmental stage, and b) segmenting the images requires rather sophisticated processing pipelines [6,9,10]. Light curtain arrays (LC) are a recently introduced phenotyping technology, which has been used successfully to assess canopy height in the field [11,12]. The setup consists of a pair of parallel bars, one radiating and the other receiving the emitted light (Figure 1A). In this way, the sensor records whether or not the light beams are interrupted by an object. By scanning the crop of interest, LC produce a binary data set of the plants’ profile (Figure 1B). Unlike imaging methods, the plant distance to the sensor and illumination conditions during measurement do not affect the data. For a given geometry of each emitter and receiver arrays, no calibration of the sensors is needed and considerably less data processing steps as compared to imaging methods, are required (Figure 1B–E; Figure 2). However, a systematic approach to address the potential of LC in assessing leaf area of individual plants or sets of plants in greenhouse cultivation was, to our knowledge, not previously conducted.

Phenotyping platforms may be operated using plant-to-sensor or sensor-to-plant measurement routines depending if plants are moved to the sensor or vice-versa, respectively. With the exception of small rosette plants [13-15], there are certain limitations in implementing imaging methods as a sensor-to-plant approach. Not only several cameras would need to be moved at a defined orientation above the plant, but also light conditions need to be strictly controlled during imaging. This need partly explains that in existing platforms, dedicated to phenotypic evaluation of plants of different sizes, a plant-to-sensor approach is implemented, where plants are moved (manually or automatically) to dedicated imaging stations (e.g. [6,16,17]). Contrary to this, LC can be more easily implemented using either plant-to-sensor or sensor-to-plant methodologies. For example, LC may offer an alternative in assessing large size plants (e.g., taller than 1 m) by employing different plant cultivation systems.

The aim of this study was to investigate whether or not LC can accurately estimate plant biomass in addition to maximum height (base to the highest leaf tip) in species with contrasting shoot architecture. To test the applicability of this method to field scale, we also estimated the biomass of plants spaced at different distances where increasing overlaps took place.

## Results and discussion

### A constant scanning speed is critical for estimating leaf area

The sensor positioning system was a prototype developed for shoot phenotyping. As the scanning speed determines the system throughput and the scalability of the approach to larger number of plants, we tested by recording plant silhouettes (also referred as profiles [11], see Figure 1B) whether or not it affects the acquired data in the speed range of 0.9 to 23.4 m min^−1^. The assessment was carried out in rapeseed plants covering a range of both leaf area (64–350 cm^2^) and maximum height (10–20 cm). Maximum height refers to the length from the base to the highest leaf tip (Figure 1C–E). We observed that calculated pixel area exponentially decreased as scanning speed increased (Additional file 1: Figure S1A). Instead, the calculated maximum height remained constant (Additional file 1: Figure S1B).

**Figure 3:**
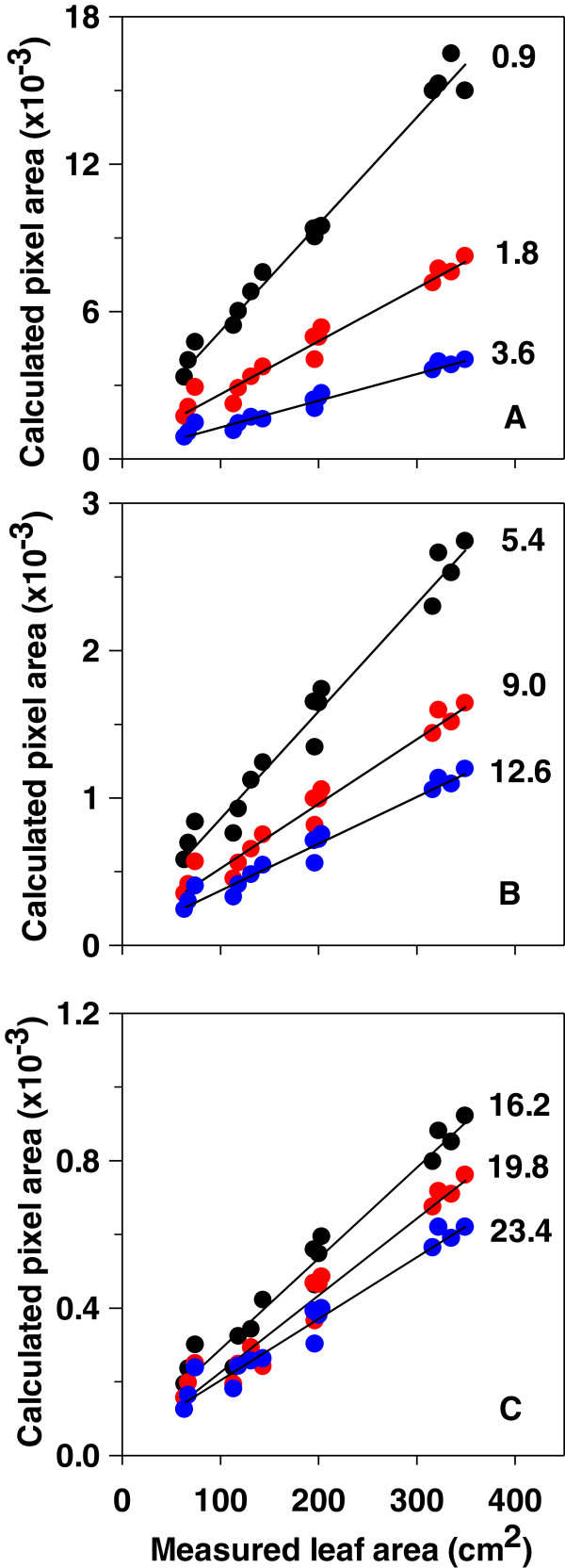
**Calculated plant pixel area (average of 18 consecutive silhouettes differing by 10°) as a function of measured plant leaf area at different scanning speeds in rapeseed.** Values in each panel indicate an increasing scanning speed during measurement and are plotted with a different symbol colour. **A** Scanning speed 0.9, 1.8, 3.6 m min^−1^. **B** Scanning speed 5.4, 9.0, 12.6 m min^−1^. **C** Scanning speed 16.2, 19.8, 23.4 m min^−1^. Correlation coefficients ranged between 0.9589 and 0.9865 (slopes all significant at P < 0.0001). Leaf area ranged between 64 and 350 cm^2^ (n = 15).

Plant silhouettes were recorded at each scanning speed for a given position (referred as starting position), as well as for 17 view angles, each differing by 10° from the previously measured one. These values were further averaged. Figure 3 shows the correlation between calculated pixel area at different scanning speeds, as described above, and leaf area measured destructively. This correlation was always highly significant (R^2^ ≥ 0.95). However, the relation between these parameters strongly depended on the scanning speed.

LC have been previously used to determine canopy height in the field [11,12]. Here we show that for maximum height estimations by using LC, a wide range of constant (0.9–23.4 m min^−1^) or even varying scanning speeds can be used without any noticeable effects on the obtained data. Instead, a constant scanning speed is essential for estimating plant leaf area. Moreover, in this case, a speed-specific relation between calculated pixel area and measured leaf area needs to be obtained in each case.

### Leaf area estimation by means of pixel area is improved as more silhouettes are averaged and/or the larger the view angle between them

Experiments were conducted in four species with contrasting shoot architecture, including two monocotyledonous (barley, maize) and two dicotyledonous (rapeseed, tomato) ones. Plants were grown from two days following transplanting up to four weeks. Because the information that can be extracted by a single silhouette may be limited due to overlapping (i.e. not visible at a given angle) plant parts, several silhouettes were recorded. At first, plant silhouette was determined at the starting position. The pot was then rotated by 10° with respect to the horizontal and the next plant silhouette was measured. Subsequently, the same procedure was repeated for 16 times, covering in total the 0 to 170° angle range.

**Figure 4:**
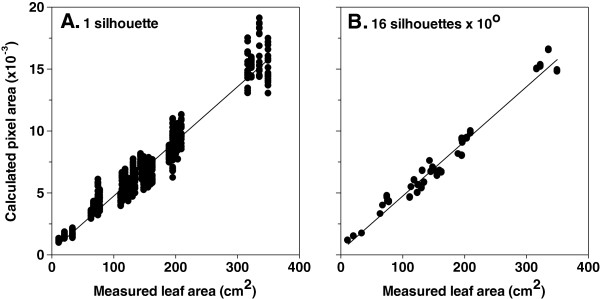
**Calculated plant pixel area by using a single silhouette (A) or averaging 16 consecutive silhouettes differing by 10° (B) as a function of measured plant leaf area in rapeseed.** Correlation coefficients were 0.9382 and 0.9717 for **A** and **B**, respectively (both slopes significant at *P* < 0.0001). Thirty five plants were assessed. Measurements were conducted at a constant scanning speed of 0.9 m min^−1^.

Figure 4 shows the correlation between all acquired silhouettes versus the calculated pixel area, where each vertical cloud of points represents a single plant. In panel A, a single plant is represented by 18 points. For panel B, three means of 16 consecutive positions (corresponding to 0–150°, 10–160° and 20–170° plant rotation) were calculated for each plant. These three means nearly overlapped (Figure 4B).

**Figure 5:**
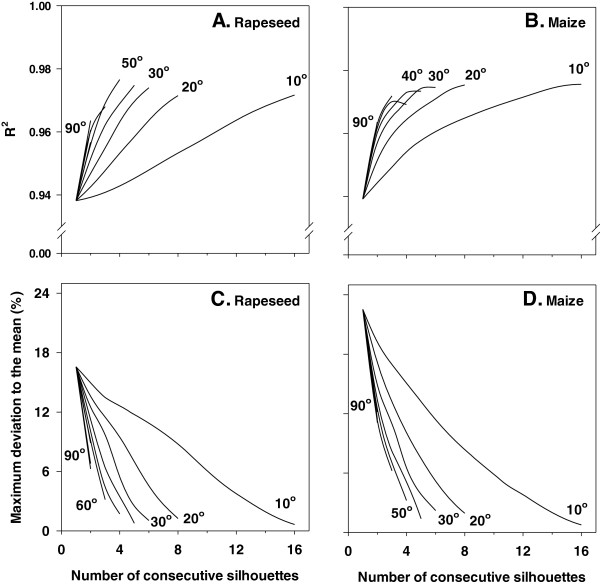
**Correlation coefficient between calculated plant pixel area versus measured plant leaf area (A, B), and the maximum deviation to the mean (expressed as percentage; C, D) as a function of number of consecutive silhouettes that are taken into account as well as the angle between them in two species.** Leaf area ranged between 12 and 350 cm^2^ for rapeseed (n = 35), and between 7 and 317 cm^2^ for maize (n = 31). Measurements were conducted at a constant scanning speed of 0.9 m min^−1^.

The effect of averaging an increasing number of consecutive profiles (1–16) as well as of using larger angles (10–90°) between consecutive profiles on the correlation coefficient between calculated plant pixel area and measured leaf area is given in Figure 5. This correlation coefficient was very high (R^2^ ≥ 0.938) when a single silhouette was employed in both rapeseed and maize, and further increased (R^2^ ≥ 0.97) when more consecutive silhouettes were averaged and/or larger angles were taken between the consecutive silhouettes (Figures 5A, B). Similar results were obtained for tomato and barley (Additional file 2: Figure S2A, B).

The maximum deviation to the mean quantifies the extent to which the calculated value of a given silhouette deviates from the average value of 16 consecutive silhouettes and expresses the maximum error that can occur. In other words, this parameter expresses the percentage of the difference of the most distant value in each vertical cloud of points (see Figure 4A), representing a single plant, with respect to the average. Although the correlation coefficient between calculated plant pixel area and measured leaf area is very high (R^2^ ≥ 0.938) when taking into account only one silhouette, the maximum deviation to the mean in calculated pixel area of a single silhouette is 17 and 23% for rapeseed and maize, respectively (Figure 5C, D). The maximum deviation to the mean rapidly decreased as more consecutive silhouettes are taken into account and/or using larger angles between them (Figure 5C, D). For instance, the maximum deviation to the mean was about 3% when four profiles differing by 50° were averaged in both studied species. The same trend was observed when analysing tomato or barley (Additional file 2: Figure S2C, D).

**Figure 6:**
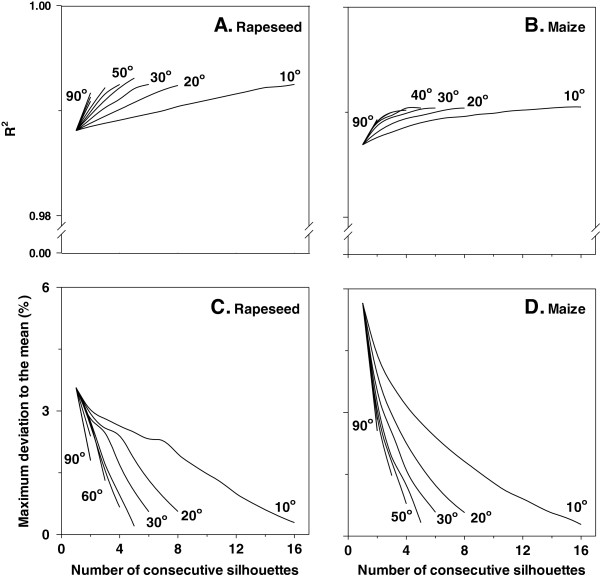
**Correlation coefficient between calculated and measured maximum plant height (base to the highest leaf tip; A, B), and the maximum deviation to the mean (expressed as percentage; C, D) as a function of number of consecutive silhouettes that are taken into account as well as the angle between them in two species.** Maximum plant height ranged between 3 and 20 cm for rapeseed (n = 35), and between 7 and 68 cm for maize (n = 31). Measurements were conducted at a constant scanning speed of 0.9 m min^−1^.

The correlation coefficient between calculated and measured maximum plant height was close to 0.99 (Figure 6A, B). This correlation coefficient was hardly affected by averaging more plant silhouettes or increasing the angles between them. Unlike leaf area estimations, for maximum plant height the maximum deviation to the mean was low (≤6%) for a single profile (Figure 6C, D). Averaging more profiles or increasing the angle between them strongly decreased the maximum deviation to the mean between calculated and measured maximum height. Analysing maximum plant height in tomato and barley gave similar results (Additional file 3: Figure S3).

Despite the high correlation coefficient (≈0.94, and 0.85 for barley) between calculated plant pixel area and measured leaf area in all four species when considering a single silhouette, we found that the maximum deviation to the mean (expressed as percentage) ranged between 17 and 29% (corresponding to rapeseed and barley, respectively). In other words, a single silhouette may result in up to 29% mis-estimation of plant pixel area, which will lead to a respective error in the calculation of leaf area. We found that this issue is readily solved by averaging an increasing number of consecutive profiles and/or using larger angles between them. We observed that the number of such consecutive profiles that needs to be averaged was not dependent on plant size within the measured range (an example is given in Additional file 4: Figure S4). For any given maximum deviation to the mean, the number of needed profiles at several angle options between them can be calculated. Plant rotation may be performed manually or automatically (by using a rotating stage). Another possibility is to use multiple pairs of LC that capture the plant profiles at different angles. By adopting this approach, several silhouettes at different angles can be recorded within a single routine that is amenable to automation.

When considering a single silhouette the correlation coefficient (approximately 0.97, and 0.83 for barley) between calculated and measured maximum height was higher in all four species compared with the one estimating leaf area. Consequently, the maximum error in the estimation of maximum height was comparatively lower (<10%, and 15% for barley) and it was reduced using the same approach, as mentioned above.

When scanning a row of (non-rotating) plants on a cultivation table in the greenhouse or in the field, one most probably will encounter a difference from the real total pixel area of these plants. Since this difference for each individual plant is sometimes positive and other times negative, most probably averaging a large number of plants will be closer to the real total pixel area than estimated by adding individual errors.

### Calculated and measured values of both maximum height and leaf area were linearly related, the latter being sensitive to shoot architecture

**Figure 7:**
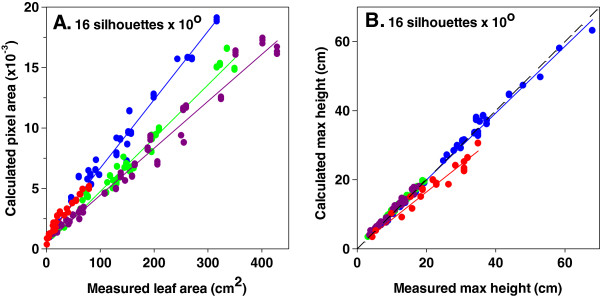
**Calculated plant pixel area (A) and calculated max height (base to the highest leaf tip; B) versus measured values in four species (maize, blue; barley, red; tomato, purple; rapeseed, green).** Correlation coefficients for all species ranged between 0.9542 and 0.9925 (*P* < 0.0001). For maximum height a single regression model was highly significant (*P* < 0.0001). For leaf area the slope of a single regression model is significant for monocyledonous species (*P* < 0.0001) but not for dicotyledonous ones (*P* = 0.34). The dashed line depicts the 1:1 relationship. Measurements were conducted at a constant scanning speed of 0.9 m min^−1^.

By plotting the calculated plant pixel area against the measured leaf area, it became apparent that the assessed dicotyledonous species (rapeseed, tomato) largely overlapped (Figure 7A). Data of the monocotyledonous species (maize, barley) showed the same trend. However, we observed that the relation between calculated plant pixel area and measured leaf area was different between monocotyledonous and dicotyledonous species (Figure 7A). Calculated pixel area was also significantly correlated with measured shoot dry weight (Additional file 5: Figure S5).

Unlike leaf area, dicotyledonous and monocotyledonous species showed similar relation between calculated and measured maximum height (Figure 7B). In all cases, calculated maximum height was the same as the one measured manually with the exception of barley for which a slight underestimation was observed for plants around 30 cm tall.

The relation between calculated pixel area and measured leaf area apparently depends on shoot architecture. Monocotyledonous and dicotyledonous species showed distinct differences in this relation. Instead, maximum height estimations were not species-dependent. In barley, an underestimation of maximum height was observed. The pixels connecting plant parts are not always continuous (Figure 1C–E). In this species missing pixels for the very thin profile of leaf tips at the position where the maximum height is estimated may account for this underestimation.

### The pixel area of several overlapping plants is indicative of total leaf area

**Figure 8:**
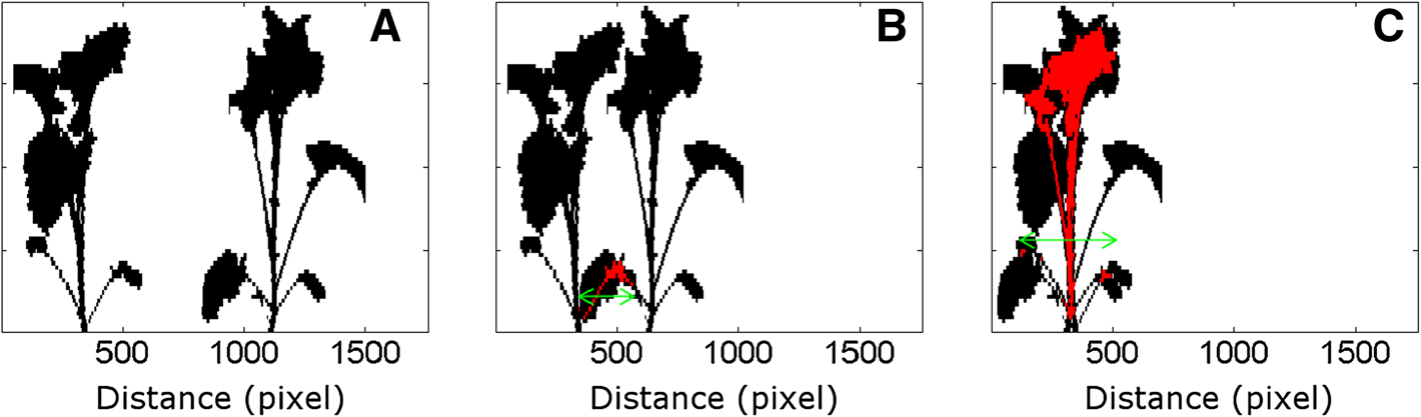
**Overlapping width (the maximum distance of any overlapping pixel in the horizontal direction), depicted by the arrow, as spacing distance decreases.** Red areas represent the overlap. **A** No overlap between silhouettes. **B** Moderate overlap between silhouettes. **C** Maximum overlap between silhouettes.

A simulation study was conducted to evaluate the effect of plant spacing distance on the calculated pixel area (an example is shown in Figure 8). Simulations were conducted using pairs of plants, spaced between 0 and 20 cm, with a 4 cm step (Additional file 6: Figure S6 and Additional file 7: Figure S7). In each spacing distance, the total pixel area (i.e. of both plants) was calculated for every view angle of both plants yielding 18 × 18 combinations. The total pixel area was then compared to the value at which the two plants were spaced at a distance where no overlapping parts occurred. Moreover, we calculated the width of the overlapping area, namely the maximum distance of any overlapping pixel in the horizontal direction. Simulations included three pairs of plants, each pair having small, medium or large leaf area (Additional file 6: Figure S6 and Additional file 7: Figure S7).

At 12 cm spacing distance, the width of the overlapping area was generally limited (≤8 cm), resulting in a very small underestimation of total pixel area (≤5%; Additional file 6: Figure S6 and Additional file 7: Figure S7). Decreasing the plant distance from 8 cm to lower values resulted in a higher width of the overlapping area, leading to increased pixel area underestimation. At the same spacing distance, both the width of the overlapping area and the pixel area underestimation increased with plant size in rapeseed and tomato (Additional file 6: Figure S6 and Additional file 7: Figure S7). Instead, the pixel area underestimation was higher in smaller maize and barley plants.

Attempting to scale this method to a typical field plant array, one may (and will) encounter overlapping parts of neighbouring plants, reducing the accuracy of estimation. This depression of pixel area, as a result of overlaps, depends on spacing distance, plant size, and shoot geometry. Spacing distance 0 cm represents a situation in which plants are placed in two different rows behind each other, and the LC scan both rows simultaneously. Scanning two rows at the same time (i.e. spacing distance 0 cm) produced the largest underestimation in all four species. Against expectations, two plants behind each other resulted in underestimation which was far less than 100%, since their structures were never identical; meaning that in none of the different orientations of a plant covered the other plant. A very small spacing distance of 4 cm resulted in less underestimation of plant pixel area, as compared to 0 cm, but the magnitude was still considerable. At 0 or 4 cm spacing distances, the larger the plant, the higher was the underestimation of pixel area in rapeseed and tomato. Contrary to these findings, the highest underestimation was observed in small maize and barley plants. At more realistic spacing distances (≥8 cm) like those used in the field in row sowing, the underestimation was rather small in all cases for the range of plant size that we examined.

## Conclusions

Light curtain arrays (LC) are a phenotyping tool yielding a binary data set of the shoot profile. It can be employed as either a sensor-to-plant (as in this study) or a plant-to-sensor system. Here we found that LC give a rapid estimate of leaf area and maximum height on individual plants of four species with contrasting shoot architecture. The accuracy of this estimation is improved as more plant profiles, following rotation, are averaged and/or the larger the angle between the acquired profiles. Leaf area estimation is strongly influenced by the scanning speed, whereas maximum height estimation is insensitive. Based on these experiments, we generally expect that up to early tillering stage for monocotyledonous species and until the 6^th^ leaf stage in dicotyledonous ones, the estimation of leaf area can be sufficiently accurate. At later developmental stages, the current method may not be necessarily suitable for precise estimation of leaf area. However, regarding measurement of maximum height, LC are only limited by the vertical (and customizable) dimension of the sensor. By using LC the biomass of several plants placed in a row can be calculated, even when those partly overlap opening the window to additional field application.

## Methods

### Plant material and growth conditions

Experiments included two dicotyledonous (*Brassica rapa* cv. Campino, *Lycopersicon esculentum* cv. Harzfeuer) and two monocotyledonous (*Zea mays* cv. Badischer Gelber, *Hordeum vulgare* cv. Barke) species. Seeds were sown, germinated, and plants were grown up to the cotyledons stage (i.e. fully open cotyledons and before the appearance of the first leaf) or the second leaf stage for the dicotyledonous and monocotyledonous species, respectively. This period ranged from twelve to fourteen days, depending on the species. Subsequently, seedlings were transplanted in 2 L pots containing commercially available soil (‘Nullerde’ Archut Erzeugnisse GmbH, Vechta, Germany), which were randomly distributed over a glasshouse compartment, located in central part of Germany (Jülich, 50.9°N). The day and night set points were 19 and 17°C for air temperature, while for relative air humidity these were 60 and 50%, respectively. Supplementary light was provided by high-pressure sodium lamps (SON-T Agro, 250 W, Philips, Eindhoven, The Netherlands) at 50 μmol m^−2^ s^−1^ photosynthetic photon flux density for 16 h per day (from 0600 to 2200 hours). This light intensity was recorded during the nocturnal period at 50 cm from the root-to-shoot interface.

The plants were watered weekly with 100 ml of a full strength Hoagland solution [18]. Two to three days following watering with Hoagland solution, plants were flushed with rainwater (approximately 10% drainage) to prevent salt accumulation. Measurements were conducted between two days and four weeks after transplanting.

### Sensor positioning system

The sensor positioning system, employed for the experiment, was a prototype developed for phenotyping purposes. It consists of five laterally movable table elements and an overhead loadbearing system. The layout of the tables enables their movement into different arrangements in which they are closer or further apart, allowing an optimal use of the available space, as explained below. During operation, the tables are automatically arranged in a way that gaps on both sides of a given table are created. In this way, it is possible for the overhead loadbearing system to scan that table, carrying the plants designated for the measuring cycle. The remaining four tables are positioned without gaps. The same principle is used to enable access for the personnel. In this way the space, which would be required for passageways between conventional greenhouse tables, is made available for experimentation. The overhead load bearing system consists of an aluminum framework, designed to minimize shadowing. It has a payload capacity of 50 kg. It can move in both x and y directions (accuracy of ± 1 mm), as well as in the z direction (accuracy of ± 2 mm). The x and y movement is due to a laterally movable crossbeam, equipped with a longitudinal carriage rail. The movement in the z direction is enabled through two cantilever arms, equipped with carriage rails. The technical conception and realization was performed by a company (MK-Maschinenbau Kitz GmbH, Troisdorf, Germany).

### LC measurements

The LC system is a parallel set of light barriers at a distance of 2.5 mm, which are arranged on two vertical poles (INFRASCAN 5000, Sitronic GmbH, Austria). The vertical support poles are 1.59 m long (1.43 m of which contain light barriers), spaced at a (horizontal) distance of 55 cm (Figure 1A). One set of light barriers is emitting infrared light (950 nm), while the other one is receiving the emitted light. In this way, the sensor detects if any of these beams are interrupted by an object placed between the emitting and receiving sides.

Before measurement, sixteen plants were placed in a row on a bench (Figure 1A). To minimize overlaps, pots were spaced at 30 cm. The light barriers were guided along the row of plants, in a way that the emitter was placed from one side of the measurement bench (perpendicular to the horizontal), while the receiver was placed on the other side (Figure 1A). The bottom edge of the light barriers was located at the bench level, allowing monitoring of both pots and plants. Care was taken that no plant parts were below the upper pot edge. All measurements took place at a constant sensor speed.

At first, the plant silhouette was recorded (Figure 1B). Subsequently, the plants were rotated by 10° to the horizontal, and another scan was conducted. In total 18 scans were acquired, starting from the initial position (0°) up to 170° with a 10° rotation for each run. Plants were not rotated by 180°, since this would result in the same silhouette as the initial position (0°). View angles larger than 180° were also not acquired, since the yielded data have been already recorded (e.g. 190° the same silhouette as 10°, 200° the same silhouette as 20°, etc.).

After all plant silhouettes were recorded, the number of leaves (≥1 cm), leaf area, leaf and plant (aboveground) dry mass were recorded. Leaf area was determined with a leaf area meter (LI-COR Model Li-3100, LI-COR, Lincoln, NE), and dry weight was assessed after drying the tissue for 24 h at 80°C.

### Plant pixel area and maximum height estimation

The LC yield a binary data set, where the values 0 and 1 correspond to continuous or interrupting light path of a given photodiode. The methodology employed to extract a single shoot silhouette from a scan of several plants is described in Figure 2. At first, individual plant position was identified from the scans including several plants (Figure 1B). This was done by first defining the pot center. Secondly, small size plant parts (e.g. stems and petioles) that were not connected to the silhouette were included (see Figure 1C–E). To identify which data belong to a given plant, a radial search within a radius of 25 pixels was conducted, beginning at the bottom center of each pot position. In this way, all pixels belonging to that area are added to the silhouette. Finally, the pixels belonging to the pot were removed from the silhouette (Figure 1B). Starting at the bottom of the silhouette, we defined that the upper edge of the pot is at the position where the number of pixels in two consecutive rows differed more than 80%. With this approach, any plant parts below the upper edge of the pot are also removed. Plant pixel area was taken as the number of pixels belonging to a given silhouette, while maximum plant height was taken as the maximum pixel value in the y-axis (Figure 1C–E). The analysis was conducted by using the MATLAB program (R2012b, MathWorks, Natick, MA).

### Simulation of spacing distance

To evaluate the potential of LC in assessing plant leaf area of overlapping plants, simulations were conducted at variable spacing distances. Two representative small, medium and large plants per species were selected. In each pair of plants, the spacing distance was decreased from 20 cm (where no overlaps occurred) down to 0 cm (maximum overlapping areas), with a step of 4 cm. In each spacing distance, simulations included all different angle combinations (18 × 18 view angles). In each spacing distance and angle combination, the pixel area of the two plants was assessed and this was compared to the sum of pixel areas of the two individual plants.

## Abbreviation

LC: Light curtain arrays.

## Competing interests

The authors declare that they have no competing interests.

## Authors’ contributions

DF performed the experimental work, acquired the silhouettes, carried out the data analysis and interpretation, and wrote the manuscript. CB developed the software tools for handling the silhouettes, and performed the simulations. JFJM and AU were responsible for the development and description of the sensor positioning system, as well as were involved in the development of the LC. SK and AP wrote the routine for sensor positioning, and were responsible for the hardware setup. FF and US supervised the study. All authors contributed in reading, editing and approving the final manuscript.

## Supplementary Material

Additional file 1: FigureS1 The effect of scanning speed on calculated plant pixel area (average of 18 consecutive silhouettes differing by 10°) and calculated maximum plant height (base to the highest leaf tip; average of 18 consecutive silhouettes differing by 10°) in rapeseed. Both are expressed as a percentage of the value at lowest scanning speed (0.9 m min^−1^). Leaf area ranged between 64 and 350 cm^2^, while maximum plant height varied between 10 and 20 cm. The SEM bars are not visible, because the SEM is smaller than the symbol (n = 15). Click here for file

Additional file 2: FigureS2 Correlation coefficient between calculated plant pixel area versus measured plant leaf area (A, B), and the maximum deviation to the mean (expressed as percentage; C, D) as a function of number of consecutive silhouettes that are taken into account as well as the angle between them in two species. Leaf area ranged between 13 and 429 cm^2^ for tomato (n = 36), and between 2 and 80 cm^2^ for barley (n = 29). Measurements were conducted at a constant scanning speed of 0.9 m min^-1^. Click here for file

Additional file 3: FigureS3 Correlation coefficient between calculated and measured maximum plant height (base to the highest leaf tip; A, B), and the maximum deviation to the mean (expressed as percentage; C, D) as a function of number of consecutive silhouettes that are taken into account as well as the angle between them in two species. Maximum plant height ranged between 4 and 18 cm for tomato (n = 36), and between 4 and 35 cm for barley (n = 29). Measurements were conducted at a constant scanning speed of 0.9 m min^-1^. Click here for file

Additional file 4: FigureS4 Maximum deviation to the mean (expressed as percentage) as a function of calculated pixel area (indicative of plant size) in two species. Six consecutive silhouettes, following plant rotation at different angles, were averaged. Leaf area ranged between 12 and 350 cm^2^ for rapeseed (n = 35), and between 7 and 317 cm^2^ for maize (n = 31). Measurements were conducted at a constant scanning speed of 0.9 m min^−1^. Click here for file

Additional file 5: FigureS5 Calculated plant pixel area versus measured dry weight in four species (maize, blue; barley, red; tomato, purple; rapeseed, green). Correlation coefficients ranged between 0.9517 and 0.9764 (slopes all significant at *P* < 0.0001). Measurements were conducted at a constant scanning speed of 0.9 m min^−1^. Click here for file

Additional file 6: FigureS6 Underestimation of calculated plant pixel area as a function of width of the overlapping area at different plant distances (referred by different colours) in two species. The width of the overlapping area refers to the maximum distance of any overlapping pixel in the horizontal direction (see Figure 8). Simulations were conducted by using a pair of small (A, B; 68 and 78/ 77 and 79 cm^2^ leaf area), medium (C, D; 201 and 210/ 153 and 158 cm^2^ leaf area) and large (E, F; 317 and 323/ 244 and 271 cm^2^ leaf area) rapeseed and maize plants, respectively. Measurements were conducted at a constant scanning speed of 0.9 m min^−1^. Click here for file

Additional file 7: FigureS7 Underestimation of calculated plant pixel area as a function of width of the overlapping area at different plant distances (referred by different colours) in two species. The width of the overlapping area refers to the maximum distance of any overlapping pixel in the horizontal direction (see Figure 8). Simulations were conducted by using a pair of small (A, B; 69 and 70/ 17 and 18 cm^2^ leaf area), medium (C, D; 189 and 194/ 34 and 39 cm^2^ leaf area) and large (E, F; 255 and 260/ 63 and 64 cm^2^ leaf area) tomato and barley plants, respectively. Measurements were conducted at a constant scanning speed of 0.9 m min^−1^. Click here for file
