# Supplementary material for: Rapid determination of leaf area and plant height by using light curtain arrays in four species with contrasting shoot architecture
Source: Plant Methods. 2014 Apr 11;10:9. doi: 10.1186/1746-4811-10-9 (PMC4022354; doi:10.1186/1746-4811-10-9)
Supplement: Additional file 1: FigureS1 — The effect of scanning speed on calculated plant pixel area (average of 18 consecutive silhouettes differing by 10°) and calculated maximum plant height (base to the highest leaf tip; average of 18 consecutive silhouettes differing by 10°) in rapeseed. Both are expressed as a percentage of the value at lowest scanning speed (0.9 m min−1). Leaf area ranged between 64 and 350 cm2, while maximum plant height varied between 10 and 20 cm. The SEM bars are not visible, because the SEM is smaller than the symbol (n = 15). [file 1746-4811-10-9-S1.docx]

**Additional file 1:Figure S1.** The effect of scanning speed on calculated plant pixel area (average of 18 consecutive silhouettes differing by 10^o^) and calculated maximum plant height (base to the highest leaf tip; average of 18 consecutive silhouettes differing by 10^o^) as a function of scanning speed in rapeseed. Both are expressed as a percentage of the value at lowest scanning speed (0.9 m min^-1^). Leaf area ranged between 64 and 350 cm^2^, while maximum plant height varied between 10 and 20 cm. The SEM bars are not visible, because the SEM is smaller than the symbol (n = 15).
