# Supplementary material for: Rapid determination of leaf area and plant height by using light curtain arrays in four species with contrasting shoot architecture
Source: Plant Methods. 2014 Apr 11;10:9. doi: 10.1186/1746-4811-10-9 (PMC4022354; doi:10.1186/1746-4811-10-9)
Supplement: Additional file 2: FigureS2 — Correlation coefficient between calculated plant pixel area versus measured plant leaf area (A, B), and the maximum deviation to the mean (expressed as percentage; C, D) as a function of number of consecutive silhouettes that are taken into account as well as the angle between them in two species. Leaf area ranged between 13 and 429 cm2 for tomato (n = 36), and between 2 and 80 cm2 for barley (n = 29). Measurements were conducted at a constant scanning speed of 0.9 m min-1. [file 1746-4811-10-9-S2.docx]

**Fig. 2.** Correlation coefficient between calculated plant pixel area versus measured plant leaf area (A, B), and the maximum deviation to the mean (expressed as percentage; C, D) as a function of number of consecutive silhouettes that are taken into account as well as the angle between them in two species. Leaf area ranged between 13 and 429 cm^2^ for tomato (n = 36), and between 2 and 80 cm^2^ for barley (n = 29). Measurements were conducted at a constant scanning speed of 0.9 m min^-1^.
