# Supplementary material for: Rapid determination of leaf area and plant height by using light curtain arrays in four species with contrasting shoot architecture
Source: Plant Methods. 2014 Apr 11;10:9. doi: 10.1186/1746-4811-10-9 (PMC4022354; doi:10.1186/1746-4811-10-9)
Supplement: Additional file 3: FigureS3 — Correlation coefficient between calculated and measured maximum plant height (base to the highest leaf tip; A, B), and the maximum deviation to the mean (expressed as percentage; C, D) as a function of number of consecutive silhouettes that are taken into account as well as the angle between them in two species. Maximum plant height ranged between 4 and 18 cm for tomato (n = 36), and between 4 and 35 cm for barley (n = 29). Measurements were conducted at a constant scanning speed of 0.9 m min-1. [file 1746-4811-10-9-S3.docx]

**Additional fie 3: Figure S3.** Correlation coefficient between calculated and measured maximum plant height (base to the highest leaf tip; A, B), and the maximum deviation to the mean (expressed as percentage; C, D) as a function of number of consecutive silhouettes that are taken into account as well as the angle between them in two species. Maximum plant height ranged between 4 and 18 cm for tomato (n = 36), and between 4 and 35 cm for barley (n = 29). Measurements were conducted at a constant scanning speed of 0.9 m min^-1^.
