# Supplementary material for: Rapid determination of leaf area and plant height by using light curtain arrays in four species with contrasting shoot architecture
Source: Plant Methods. 2014 Apr 11;10:9. doi: 10.1186/1746-4811-10-9 (PMC4022354; doi:10.1186/1746-4811-10-9)
Supplement: Additional file 4: FigureS4 — Maximum deviation to the mean (expressed as percentage) as a function of calculated pixel area (indicative of plant size) in two species. Six consecutive silhouettes, following plant rotation at different angles, were averaged. Leaf area ranged between 12 and 350 cm2 for rapeseed (n = 35), and between 7 and 317 cm2 for maize (n = 31). Measurements were conducted at a constant scanning speed of 0.9 m min−1. [file 1746-4811-10-9-S4.docx]

**Fig. 4.** Maximum deviation to the mean (expressed as percentage) as a function of calculated pixel area (indicative of plant size) in two species. Six consecutive silhouettes, following plant rotation at different angles, were averaged. Leaf area ranged between 12 and 350 cm^2^ for rapeseed (n = 35), and between 7 and 317 cm^2^ for maize (n = 31). Measurements were conducted at a constant scanning speed of 0.9 m min^-1^.
