# Supplementary material for: Rapid determination of leaf area and plant height by using light curtain arrays in four species with contrasting shoot architecture
Source: Plant Methods. 2014 Apr 11;10:9. doi: 10.1186/1746-4811-10-9 (PMC4022354; doi:10.1186/1746-4811-10-9)
Supplement: Additional file 5: FigureS5 — Calculated plant pixel area versus measured dry weight in four species (maize, blue; barley, red; tomato, purple; rapeseed, green). Correlation coefficients ranged between 0.9517 and 0.9764 (slopes all significant at P < 0.0001). Measurements were conducted at a constant scanning speed of 0.9 m min−1. [file 1746-4811-10-9-S5.docx]

**Additional file 5: Figure S5.** Calculated plant pixel area versus measured dry weight in four species (maize, blue; barley, red; tomato, purple; rapeseed, green). Correlation coefficients ranged between 0.9517 and 0.9764 (slopes all significant at *P* < 0.0001). Measurements were conducted at a constant scanning speed of 0.9 m min^-1^.
