# Supplementary material for: Rapid determination of leaf area and plant height by using light curtain arrays in four species with contrasting shoot architecture
Source: Plant Methods. 2014 Apr 11;10:9. doi: 10.1186/1746-4811-10-9 (PMC4022354; doi:10.1186/1746-4811-10-9)
Supplement: Additional file 6: FigureS6 — Underestimation of calculated plant pixel area as a function of width of the overlapping area at different plant distances (referred by different colours) in two species. The width of the overlapping area refers to the maximum distance of any overlapping pixel in the horizontal direction (see Figure 8). Simulations were conducted by using a pair of small (A, B; 68 and 78/ 77 and 79 cm2 leaf area), medium (C, D; 201 and 210/ 153 and 158 cm2 leaf area) and large (E, F; 317 and 323/ 244 and 271 cm2 leaf area) rapeseed and maize plants, respectively. Measurements were conducted at a constant scanning speed of 0.9 m min−1. [file 1746-4811-10-9-S6.docx]

**Additional file 6: Figure S6.** Underestimation of calculated plant pixel area as a function of width of the overlapping area at different plant distances (referred by different colours) in two species. The width of the overlapping area refers to the maximum distance of any overlapping pixel in the horizontal direction (see Fig. 8). Simulations were conducted by using a pair of small (A, B; 68 and 78/ 77 and 79 cm^2^ leaf area), medium (C, D; 201 and 210/ 153 and 158 cm^2^ leaf area) and large (E, F; 317 and 323/ 244 and 271 cm^2^ leaf area) rapeseed and maize plants, respectively. Measurements were conducted at a constant scanning speed of 0.9 m min^-1^.
