# Supplementary material for: Rapid determination of leaf area and plant height by using light curtain arrays in four species with contrasting shoot architecture
Source: Plant Methods. 2014 Apr 11;10:9. doi: 10.1186/1746-4811-10-9 (PMC4022354; doi:10.1186/1746-4811-10-9)
Supplement: Additional file 7: FigureS7 — Underestimation of calculated plant pixel area as a function of width of the overlapping area at different plant distances (referred by different colours) in two species. The width of the overlapping area refers to the maximum distance of any overlapping pixel in the horizontal direction (see Figure 8). Simulations were conducted by using a pair of small (A, B; 69 and 70/ 17 and 18 cm2 leaf area), medium (C, D; 189 and 194/ 34 and 39 cm2 leaf area) and large (E, F; 255 and 260/ 63 and 64 cm2 leaf area) tomato and barley plants, respectively. Measurements were conducted at a constant scanning speed of 0.9 m min−1. [file 1746-4811-10-9-S7.docx]

**Additional file 7: Figure S7.** Underestimation of calculated plant pixel area as a function of width of the overlapping area at different plant distances (referred by different colours) in two species. The width of the overlapping area refers to the maximum distance of any overlapping pixel in the horizontal direction (see Fig. 8). Simulations were conducted by using a pair of small (A, B; 69 and 70/ 17 and 18 cm^2^ leaf area), medium (C, D; 189 and 194/ 34 and 39 cm^2^ leaf area) and large (E, F; 255 and 260/ 63 and 64 cm^2^ leaf area) tomato and barley plants, respectively. Measurements were conducted at a constant scanning speed of 0.9 m min^-1^.
